# The Movement Imagery Questionnaire-Revised, Second Edition (MIQ-RS) Is a Reliable and Valid Tool for Evaluating Motor Imagery in Stroke Populations

**DOI:** 10.1155/2012/497289

**Published:** 2012-02-28

**Authors:** Andrew J. Butler, Jennifer Cazeaux, Anna Fidler, Jessica Jansen, Nehama Lefkove, Melanie Gregg, Craig Hall, Kirk A. Easley, Neeta Shenvi, Steven L. Wolf

**Affiliations:** ^1^Department of Rehabilitation Medicine, Division of Physical Therapy, Emory University School of Medicine, Emory University, 1441 Clifton Road NE, Suite 170, Atlanta, GA 30322, USA; ^2^Atlanta Veterans Affairs Medical Center, Decatur, GA 30033, USA; ^3^Department of Kinesiology and Applied Health, The University of Winnipeg, Winnipeg, MB, Canada R3B2E9; ^4^School of Kinesiology, Western University, London, ON, Canada N6A 3K7; ^5^Department of Biostatistics and Bioinformatics, Rollins School of Public Health, Emory University, Atlanta, GA 30322, USA

## Abstract

Mental imagery can improve motor performance in stroke populations when combined with physical therapy. Valid and reliable instruments to evaluate the imagery ability of stroke survivors are needed to maximize the benefits of mental imagery therapy. The purposes of this study were to: examine and compare the test-retest intra-rate reliability of the Movement Imagery Questionnaire-Revised, Second Edition (MIQ-RS) in stroke survivors and able-bodied controls, examine internal consistency of the visual and kinesthetic items of the MIQ-RS, determine if the MIQ-RS includes both the visual and kinesthetic dimensions of mental imagery, correlate impairment and motor imagery scores, and investigate the criterion validity of the MIQ-RS in stroke survivors by comparing the results to the KVIQ-10. Test-retest analysis indicated good levels of reliability (ICC range: .83–.99) and internal consistency (Cronbach **α**: .95–.98) of the visual and kinesthetic subscales in both groups. The two-factor structure of the MIQ-RS was supported by factor analysis, with the visual and kinesthetic components accounting for 88.6% and 83.4% of the total variance in the able-bodied and stroke groups, respectively. The MIQ-RS is a valid and reliable instrument in the stroke population examined and able-bodied populations and therefore useful as an outcome measure for motor imagery ability.

## 1. Introduction

 Mental practice is used as a therapeutic intervention to improve motor performance [1]. Motor imagery, a subset of mental practice, emphasizes mental rehearsal of motor skills to improve function [1]. Mental imagery, when combined with physical or occupational therapy, has been demonstrated to improve motor performance in healthy individuals and in acute and chronic stroke populations [2–7]. Page and colleagues [5] suggest that mental practice activates the same neural networks as physical performance, providing supplemental task practice that leads to greater functional improvements than physical practice alone. Regardless of motor impairment following stroke, persons may still have the ability to mentally rehearse tasks to decrease impairment and improve function [8]. Theoretically, to benefit from motor imagery therapy, stroke survivors must be able to imagine. Therefore, appropriate evaluation tools to assess imagery ability of stroke survivors are essentially prior to implementation of mental imagery therapy in a clinical setting.

 There are motor imagery questionnaires currently available to quantify an individual's ability to perform a mental practice task. The Movement Imagery Questionnaire (MIQ) [9] and the Movement Imagery Questionnaire-Revised (MIQ-R) [10] are mental imagery questionnaires intended for implementation in healthy adult and athletic populations and include movements that require high degrees of skill and coordination. Hall and Pongrac [9] first assessed the reliability of the MIQ, an 18-item questionnaire, in fifty able-bodied individuals (mean age, 21 years). Test-retest reliability measured over one week found the questionnaire reliable, with Pearson's correlation coefficients of  .83 for both visual and kinesthetic subscales. The MIQ showed internal consistency with Cronbach's *α* values of  .87 and  .91 for both visual and kinesthetic, respectively [9]. In an effort to minimize the time needed to administer the MIQ and remove physically demanding tasks, the questionnaire was shortened from its original eighteen items to eight and renamed the MIQ-R [10]. The results of the MIQ-R were compared to that of the MIQ in fifty subjects (mean age, 21 years; range, 18–41 years) to establish criterion validity. A correlation coefficient of  −.77 (negative due to the reversal of the MIQ-R rating scale) showed the MIQ-R to be a suitable replacement [10]. The MIQ and MIQ-R have only been validated in able-bodied individuals and are inappropriate questionnaires for people with physical limitations as they include whole body movements and jumping tasks.

 The Kinesthetic and Visual Imagery Questionnaire (KVIQ) is an assessment tool designed specifically for persons with physical disabilities [11]. The KVIQ-20 and the KVIQ-10 were developed based upon a perceived need to establish an outcome measure appropriate for stroke survivors [11] and have been deemed valid and reliable in stroke, able-bodied, and age-matched populations.

 Based upon feedback from end-users, the Mental Imagery Questionnaire-Revised, Second Edition (MIQ-RS) [12] was designed to measure imagery ability in people with restricted mobility. As an adaption from the MIQ-R, the MIQ-RS was examined by Gregg and colleagues [12] in a study that investigated the reliability and validity of the MIQ-RS in a group of 320 able-bodied college-aged athletes. No significant differences were found when the MIQ-RS was compared to Hall and Martin's MIQ-R for criterion validity. Internal consistency was found to be high, with a visual score of  .87 and kinesthetic score of  .90. Test-retest reliability was also high, with a visual score of  .83 and kinesthetic score  .73 [12].

 Both the MIQ-RS and KVIQ were specifically designed to assess imagery ability in stroke populations. All of the tasks on each questionnaire are performed while sitting and are therefore relatively safe and not physically demanding. However, the KVIQ does present with some limitations. The relationship between tasks on the questionnaire and functional relevance requires further clarification. Tasks in the KVIQ are less functional and are not described in detail to the participant in advance of completing the task. The performance of active functional tasks with extensive practice has been shown to induce recovery of motor skills and abilities [13]. The MIQ-RS was designed with this perspective in mind and includes functional tasks which may better reflect an individual's motor performance and functional recovery when compared to gross, less functional movement tasks alone. In addition, the MIQ-RS is designed in such a manner (i.e., each movement is described in detail and physically executed before being imaged) that researchers employing the instrument can be confident that all participants are rating exactly the same movements (rather than variations of these movements) [14]. Though the MIQ-RS is reliable and valid in able-bodied individuals, the questionnaire has yet to be assessed in acute and chronic stroke and elderly populations.

 The present study attempts to establish the reliability and validity of the MIQ-RS in the stroke and elderly populations. The purposes of this study were to: (1) examine and compare the test-retest reliability of the MIQ-RS in stroke survivors and able-bodied, age-matched controls, (2) examine the internal consistency of the visual and kinesthetic items of the MIQ-RS, (3) use factor analysis to determine if the MIQ-RS includes both the visual and kinesthetic dimensions of mental imagery, (4) correlate impairment and motor imagery scores to provide predictive value of performance on the MIQ-RS within the stroke population, and (5) investigate the criterion validity of the MIQ-RS in stroke survivors by comparing the results to the KVIQ-10. If deemed valid and reliable, the MIQ-RS may be a useful tool to evaluate outcomes and aid in the delineation of those stroke survivors best suited for mental imagery training.

## 2. Methods

### 2.1. Participants and Design

 The MIQ-RS data from the Gregg et al. 2010 [12] study involving young able-bodied individuals were used for sample size calculations. Based on the formula for the confidence interval provided by Bland and Altman [15], approximately 20–25 participants would enable estimation of the repeatability to within 0.5 standard deviations. The study included 23 stroke survivors and 23 able-bodied people who were evaluated twice by the same evaluator within a 2-week interval. The participants with stroke were recruited from an outpatient rehabilitation program, and the able-bodied volunteers were recruited from the local population via email. All volunteers gave informed written consent as approved by the local institution review board.

**Inclusion Criteria**

Volunteers in the stroke group had persistent hemiparesis secondary to a stroke. Inclusion criteria were (1) between the ages of 40 and 80; (2) medically stable with sitting balance control; (3) chronic stroke, defined as more than 2 months but less than one hundred-sixty months prior to the study; (4) no prior mental imagery intervention; (5) not receiving physical or occupational therapy; (6) no severe aphasia, apraxia, or other neurological conditions. Persons were excluded if they had pain or range of motion limitations that would interfere with their ability to complete the questionnaires. Participants in the able-bodied group were between 40–77 years of age and reported no known physical or cognitive impairment.

Three properties of the MIQ-RS were studied. First, a single therapist repeated the questionnaires for each volunteer within a 2-week interval to investigate the test-retest reliability of the MIQ-RS and the KVIQ-10. Second, the internal consistency of the visual and kinesthetic subscales of the MIQ-RS was examined. Third, the criterion validity of the MIQ-RS in stroke survivors was tested by comparing the results to the KVIQ-10.

### 2.2. Procedure

 Fifty-two participants were recruited for this study. Participants who violated protocol or exhibited data points that were predefined as outliers were removed from further analysis. We operationally defined outliers *a priori* as data points that lie outside 1.5 times the interquartile range from the first or third quartile [16]. The KVIQ-10 was chosen as the criterion test because of its established reliability and validity within the stroke population [11], and the test duration is comparable to the MIQ-RS. The order of administration of the questionnaires was randomized to minimize any ordering effect. An independent examiner who was a doctoral-level physical therapist administered the upper extremity portion of the Fugl-Meyer Motor Assessment [17] (FMA) and Mini-Mental Status Exam (MMSE) [18] tests to the participants with stroke to establish degree of motor impairment and cognitive status, respectively. Correlation between scores on the MIQ-RS and degree of impairment and/or cognitive status was examined to provide predictive value of performance on the MIQ-RS within the stroke population.

 A complete description of the MIQ-RS and KVIQ-10 including the methods, properties, and instructions for scoring has been reported previously [11, 12]. Briefly, the procedure was as follows. The evaluator, after demonstrating the movements, asked each participant to: (1) assume the start position demonstrated by the evaluator; (2) perform the movement once with the less affected limb; (3) return to the start position and imagine the movement previously performed on the opposite limb (MIQ-RS) or the same limb (KVIQ-10); finally (4) rate the ease/difficulty of seeing and feeling (MIQ-RS) or the clarity and intensity (KVIQ-10) of the imagined movement.

### 2.3. Measurements

#### 2.3.1. Movement Imagery Questionnaire-Revised for Stroke (MIQ-RS)

 The MIQ-RS [12] is a 14-item questionnaire that rates one's ability to imagine. The questionnaire consists of 7 visual and 7 kinesthetic items and requires 25–30 minutes to administer. The tasks performed and imagined include functional and gross movements and are all performed from a sitting position. Note that the starting position in this study (i.e., sitting) is different from the starting position of Gregg et al., 2010 (i.e., standing). After imagining the movements, the participants use a seven-point Likert scale to rate the ease or difficulty of seeing and feeling the movements. A score of 1 represents “very hard to see/feel,” and a score of 7 represents “very easy to see/feel.” The MIQ-RS was administered by a trained examiner who read the instructions and recorded all scores.

#### 2.3.2. Kinesthetic and Visual Imagery Questionnaire-10 (KVIQ-10)

 The KVIQ-10 [11] is a mental imagery questionnaire that requires 20 minutes to administer and includes 5 visual and 5 kinesthetic items. This questionnaire consists of many gross movements performed while sitting (i.e., knee extension, shoulder elevation, etc.). The KVIQ-10 asks one to rate the clarity of the imagined movements (visual subscale) and the intensity of the sensations (kinesthetic subscale) using a 5-point Likert scale. A score of 5 corresponds to the highest clarity or intensity, and a score of 1 corresponds to the lowest clarity or intensity. The KVIQ-10 is also administered by a trained examiner who reads all instructions and records the scores.

#### 2.3.3. Fugl-Meyer Motor Assessment (FMA)

 The FMA [17] is a tool that assesses reflex activity, coordination, and voluntary movement in and out of synergy patterns. Thirty-three individual items are rated on a 3-point ordinal scale (0 to 2), with a maximum possible score of 66 for upper extremity motor function. A lower score indicates a higher degree of impairment.

#### 2.3.4. Mini Mental Status Examination (MMSE)

 The MMSE [18] was administered to all participants with stroke to gauge cognitive status. This tool is an 11-item measure that tests orientation, registration, attention and calculation, recall, and language. The maximum score on the MMSE is 30, with higher scores reflecting higher levels of cognition. A score of 27 or above is considered normal, with scores of 26 or below indicating varying degrees of dementia.

### 2.4. Data Analysis

 All analyses were performed using nondirectional, two-sided tests with a minimal level of significance set at *α* =  .05 using SPSS Statistics Version 17.0 (SPSS Inc., Chicago, IL USA).

#### 2.4.1. Test-Retest Reliability

 The difference between the first and second measurements was calculated for each participant, and the mean difference was used to estimate the bias between the two measurements made by the participant. The Bland-Altman [15] plot was used to visualize the agreement of the scores across sessions by plotting the difference in scores between testing sessions against the mean score for each participant. The data points should lie between 1.96 standard deviations of the mean. A coefficient of repeatability, which is an indication of the maximum difference, that is likely to occur between two measurements if there is no bias, was calculated by multiplying the standard deviation of the differences by 1.96 [15]. High repeatability corresponds with a small value for the coefficient of repeatability. We expect 95% of differences to be less than two standard deviations according to the definition of a repeatability coefficient adopted by the British Standards Institution [19]. A paired sample *t*-test was used to compare the mean differences between the first and second administrations. Reliability was assessed by calculating an intraclass correlation coefficient (ICC type 2,1), a random effects model described by Shrout and Fleiss [20] to examine the reliability of the individual items for the visual and kinesthetic subscales of the MIQ-RS. ICC values were classified as weak (0.1–0.3), moderate (0.3–0.5), or strong (>0.5) [21].

#### 2.4.2. Internal Consistency

 To determine consistency of the individual items of the visual and kinesthetic subscales of the MIQ-RS, Cronbach's *α* coefficients were computed. These coefficients were computed for the able-bodied control group, the stroke group, and both groups combined. A Cronbach *α* coefficient greater than 0.7 is generally considered acceptable [22].

#### 2.4.3. Factor Analysis

 Factor analysis was used to determine if the MIQ-RS includes both the visual and kinesthetic dimensions of mental imagery. Two-factor analyses (able-bodied only and stroke only) were performed using the data from the first administration. The principal factor extraction technique with varimax rotation was used to assess the bifactorial structure of the MIQ-RS. The varimax rotation was used to minimize the complexity of the loadings within each factor and to represent the clearest factor structure.

#### 2.4.4. Criterion Validity

 The scores on the MIQ-RS were compared to the scores on the KVIQ-10 using repeated measures analyses to determine differences on the visual and kinesthetic subscales of the questionnaires [11]. The Spearman's rank correlation coefficient was used to determine the association between scores on the MIQ-RS and the KVIQ-10 in the able-bodied control and stroke groups [23]. The choice of the Spearman rank correlation coefficient was based upon the need to avoid rescaling (i.e., the KVIQ-10 uses a 1–5 scale, while the MIQ-RS employs a scale of 1–7).

## 3. Results

**Figure 1:**
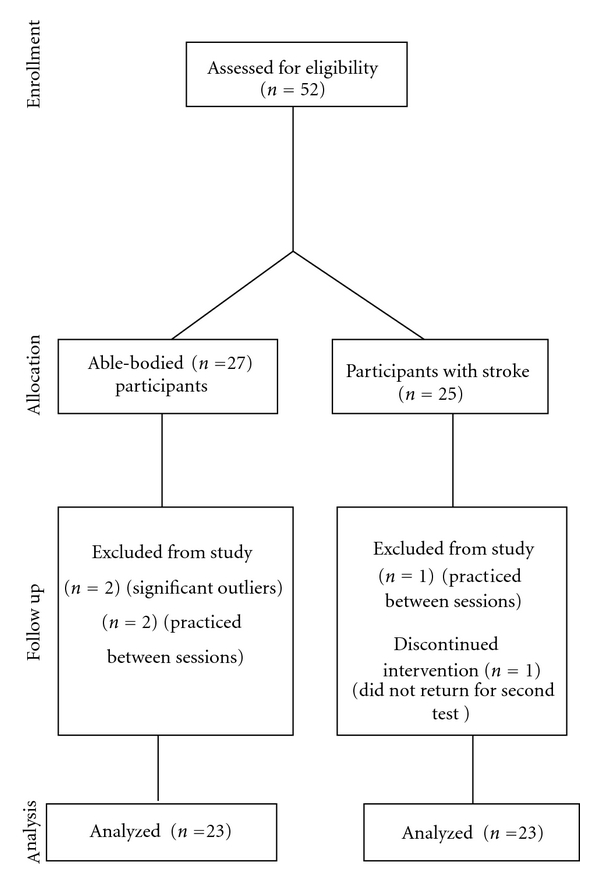
Consort diagram; flow of participants through each stage of the study.

 A consort diagram shows the flow of participants through each stage of the study (Figure 1). All volunteers were 100 percent compliant with evaluation procedures. Two members of the able-bodied control group were excluded from the analyses because the average difference of visual scores between the first and second administration classified them as outliers. Participants who practiced mental imagery (2 able-bodied participants and 1 stroke participant) between sessions were also eliminated from the data analysis due to protocol violation. One participant with stroke did not return for the second testing session and was thus removed from the study. Consequently data from 46/52 participants are reported.

 Demographic information and baseline data are presented as descriptive statistics (Table 1). The able-bodied control group was comprised of twelve males and eleven females (mean age, 51 ± 9.6 years). Sixteen males and seven females were enrolled into the stroke group (mean age, 59 ± 10.2 years). The difference in mean age between the able-bodied control and stroke group was significant (*P* = 0.008). Gender and hand dominance differences between the able-bodied and stroke groups were not statistically significant (*P* = 0.24 and *P* = 1.0, resp.).

### 3.1. Test-Retest Reliability

 There was no significant difference between scores measured at the first and second administrations of the MIQ-RS for both subscales and groups, indicating significant reliability of the instrument over time. The mean difference between test sessions and ICCs for each subscale and group is presented in Tables 2 and 3. ICCs were calculated to determine the reliability of individual items on the MIQ-RS. In the able-bodied control group, the ICCs ranged from  .64 to  .91 for the kinesthetic items and  .89 to  .95 for the visual items (Table 2). The ICC for the average kinesthetic scores across time in the able-bodied control group was  .94 with a 95% confidence interval (CI) of  .86 to  .97 (*P* = 0.51). The ICC for the average visual scores in the able-bodied control group between sessions was  .99 (.98 to  .99 95% CI) (*P* = 0.074). The ICCs ranged from  .73 to  .89 for the kinesthetic items and  .54 to  .80 for the visual items in the stroke group (Table 3). When comparing the average scores between administrations, the ICC was  .92 (.83–.97 95% CI) for the kinesthetic subscale (*P* = 0.58) and  .83 (.64–.92 95% CI) for the visual subscale (*P* = 0.96).

**Figure 2:**
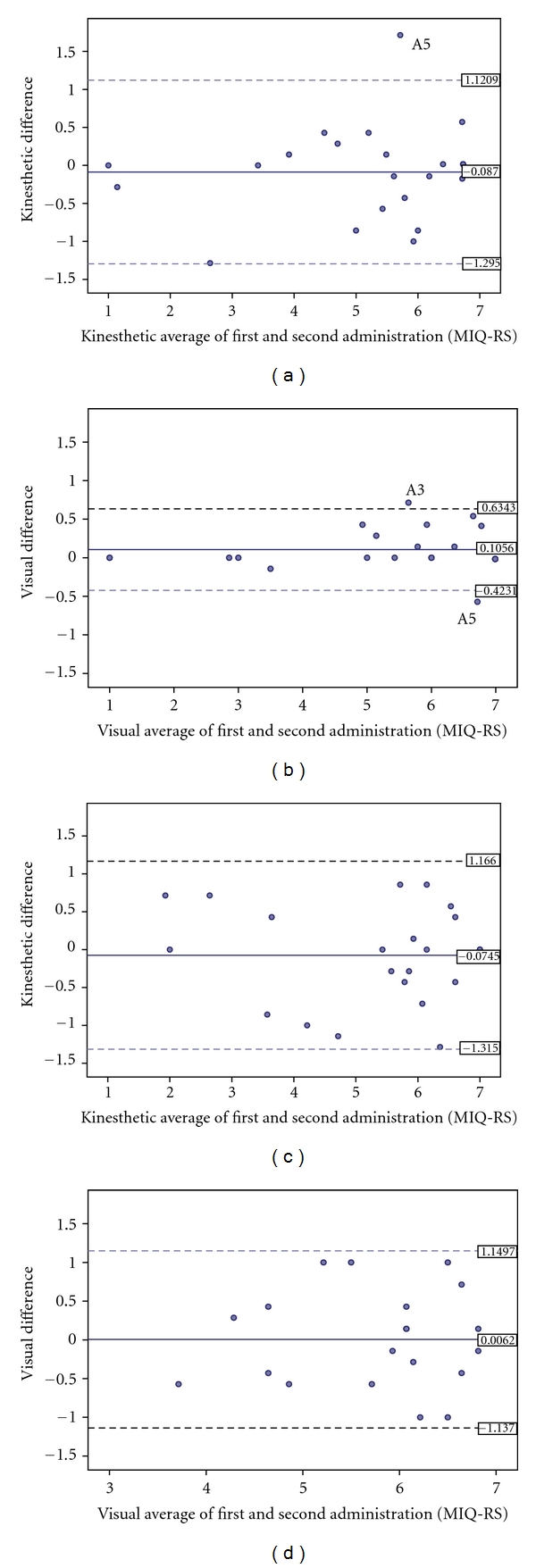
Bland-Altman plots illustrating the agreement of kinesthetic and visual scores of the MIQ-RS between sessions for individual participants. (a) kinesthetic agreement in the able-bodied group; (b) Visual agreement in the able-bodied group; (c) kinesthetic agreement in the stroke group; (d) Visual agreement in the stroke group. Horizontal lines are drawn at the mean difference, and at the limits of agreement, which are defined as the mean difference plus and minus 1.96 times the standard deviation of the differences.

 The Bland-Altman plots indicate the repeatability of the visual and kinesthetic scores of MIQ-RS between sessions (Figure 2). For the able-bodied control group, any two MIQ-RS kinesthetic scores on the same participants can differ by as much as 1.21 (Table 4—coefficient of repeatability). In other words, the difference between two MIQ-RS kinesthetic scores is expected to be less than 1.21 for 95% of all replicate pairs, while the difference between MIQ-RS visual scores is expected to be less than 0.527 for 95% of all replicate pairs. For the stroke group, the coefficients of repeatability were 1.24 for the kinesthetic scores and 1.14 for the visual scores (Table 4).

### 3.2. Internal Consistency

 Cronbach's alpha values were computed for the first and second administrations of the MIQ-RS in the able-bodied control and stroke groups or the combination of both groups (Table 5). For the first administration of the MIQ-RS, the Cronbach alpha values for the kinesthetic items were  .97 and ranged from  .95 to  .98 for the visual items. Similar Cronbach alpha values were calculated on the second administration of the test, with values of  .98 for the kinesthetic subscale and ranging from  .95 to  .98 for the visual subscale, suggesting consistency of the individual items of the visual and kinesthetic subscales of the MIQ-RS.

### 3.3. Factor Analysis

 Two components (visual and kinesthetic) were extracted for each factor analysis performed confirming the bifactorial structure of the MIQ-RS. All fourteen items of the MIQ-RS were well defined by the factor solution. Communalities ranged from 0.72 to 0.96 in the able-bodied group and 0.72 to 0.95 in the stroke group. In the able-bodied group, the two factors explained 88.6% of the total variance (component 1: 74.6% and component 2: 14.0%) and the correlation between the visual and kinesthetic components was 0.69 (*P* < 0.001). In the stroke group, the two factors explained 83.4% of the total variance (component 1: 66.6% and component 2: 16.8%), and the correlation between the visual and kinesthetic components was 0.61 (*P* < 0.001).

### 3.4. Criterion Validity

**Figure 3:**
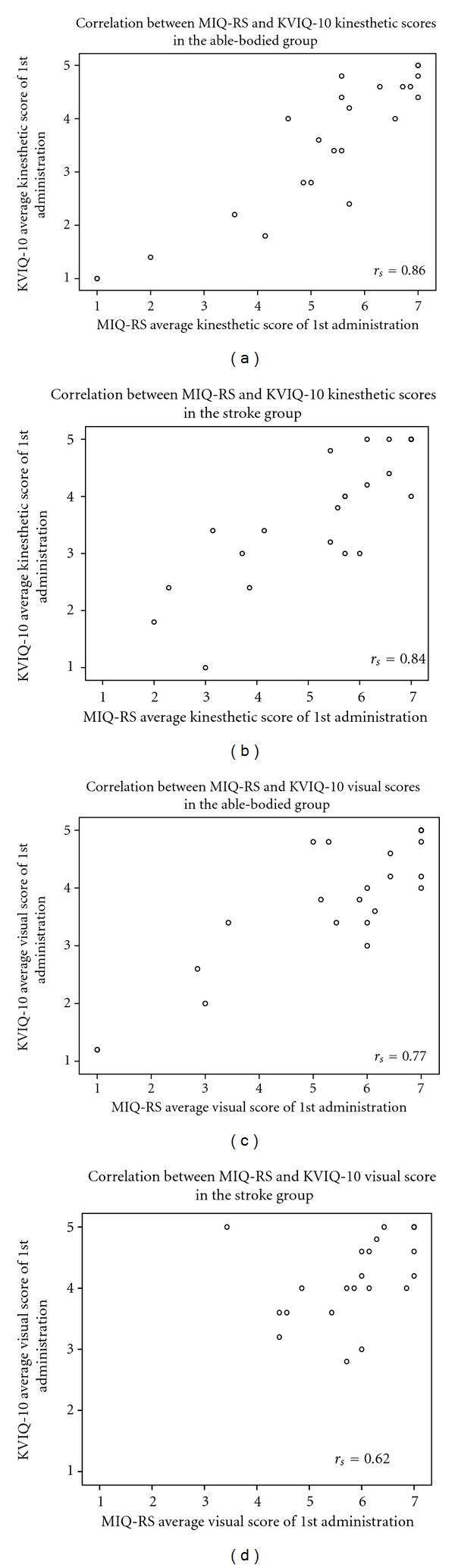
Spearman's rank correlation coefficient (*r*_*s*_) between MIQ-RS and KVIQ-10. (a) kinesthetic scores in able-bodied group; (b): kinesthetic scores in stroke group; (c): visual scores in able-bodied group; (d): visual scores in stroke group.

 The Spearman rank correlation coefficient was used to determine the association between scores on the MIQ-RS and the KVIQ-10 in the able-bodied control and stroke groups. The regression of KVIQ-10 on MIQ-RS supported criterion validity of the MIQ-RS (the slope and intercept were not significantly different from 1 and 0). This is illustrated graphically for each subscale: the kinesthetic able-bodied group Figure 3(a) (*r*_*s*_ = 0.86, *P* < 0.001), kinesthetic stroke group Figure 3(b) (*r*_*s*_ = .84, *P* < 0.001), visual able-bodied group Figure 3(c) (*r*_*s*_ = .77, *P* < 0.001), and visual stroke group Figure 3(d) (*r*_*s*_ = 0.62, *P* = 0.002).

### 3.5. Correlation between the MIQ-RS, FMA, and MMSE

 The FMA and MMSE were administered to the stroke group to determine the degree of motor impairment and cognitive status. The FMA scores ranged from 4–66 (average 32.7 ± 23), and the MMSE scores ranged from 25–30 (average 28.7 ± 1.5). Spearman's rank correlation coefficients were computed to establish potential predictive relationships between scores on the FMA and MMSE and performance on the MIQ-RS (Table 6). Correlations between kinesthetic, visual, and total scores of the MIQ-RS and the FMA upper extremity motor score were 0.40 (*P* = 0.86), 0.24 (*P* = 0.28), and 0.11 (*P* = 0.61), respectively. Correlations between kinesthetic, visual, and total scores on the MIQ-RS and the MMSE yielded similar results with values of −0.32 (*P* = 0.14), −0.037 (*P* = 0.87), and −0.24 (*P* = 0.26), respectively. These rather poor correlations suggest that the degree of motor and cognitive impairment in this particular dataset is not associated with motor imagery ability.

## 4. Discussion

 The present study demonstrates that the MIQ-RS is valid and reliable and is therefore a useful outcome measure to assess motor imagery ability in stroke and able-bodied populations. The MIQ-RS has previously been found to be valid and reliable in young able-bodied individuals [12], but this is the first study to demonstrate the reliability and validity of the MIQ-RS in a stroke population and middle-to-older individuals.

 Results from the test-retest analysis showed that the MIQ-RS is reliable over time (*P* < 0.05). The ICC values for individual visual items in the able-bodied control group demonstrated good reproducibility with values ranging from  .89 to  .95. Individual kinesthetic items showed greater dispersion of ICCs ranging from  .64 to  .91. In the stroke group, visual scores showed lower values and greater dispersion of ICCs ranging from  .54 to  .80, while kinesthetic scores showed greater stability ranging from  .73 to  .89. These findings contrast with results from previous studies [11, 12] reporting higher ICCs for visual scores than kinesthetic scores. The inverse relationship between the groups' visual and kinesthetic ICCs cannot be accounted for by individual participant characteristics as there was no statistically significant difference in demographic information (with the exception of age) between the able-bodied control and stroke groups. The explanation for this discrepancy therefore remains unclear.

 While internal consistency and factor analysis revealed that visual and kinesthetic subscales are two separate components of the MIQ-RS, they also share similar qualities as indicated by their correlation coefficients. Compared to the KVIQ-10, the MIQ-RS has shown similar internal consistency. Cronbach's alpha coefficients reported by Malouin et al. 2010 were 0.89 (visual) and 0.87 (kinesthetic) for the KVIQ-10 and  .94 (visual) and  .92 (kinesthetic) for the KVIQ-20 [11]. The variance from the data in this study explained by factor analysis (67.7%) for the KVIQ-10 was lower than reported for the MIQ-RS (*α* < 0.90; 88.6% and 83.4% for able-bodied and stroke groups). These numbers suggest less variability between items within each subscale on the MIQ-RS compared to the KVIQ-10. Therefore, for this particular dataset the MIQ-RS was the questionnaire that most accurately defined the two constructs of mental imagery.

 Although the age range for both the able-bodied control and stroke group was 40–80 years, the mean age of the stroke group (59.2 yrs) was statistically different (i.e., higher) than the mean age of the able-bodied control group (51 yrs). The age discrepancy between the able-bodied control and stroke groups may be viewed as a limitation of this study. However, further analysis revealed that age and scores on the MIQ-RS do not correlate. The correlation coefficients comparing participant age to kinesthetic and visual scores on the MIQ-RS in the able-bodied group were  .13 (*P* = 0.56) and −.18 (*P* = 0.42), respectively. Similarly, correlation between age and kinesthetic and visual scores in the stroke group were  .06 (*P* = 0.78) and −.005 (*P* = 0.98), respectively. These correlation coefficients are not statistically significant, indicating that age does not predict ability to imagine movements visually or kinesthetically. In support of this contention, Malouin and colleagues [11] found that age did not affect the reliability of the KVIQ-10 in a study involving able-bodied individuals and persons with movement limitations including stroke.

 Limitations of the current study include a low, but statistically significant, correlation (*r*_*s*_ = 0.61,  *P* = 0.002) between the MIQ-RS and KVIQ-10 visual subscales in the stroke population.

The administration of the KVIQ-10 restricts the participant to first-person imagery of movements. The MIQ-RS was designed to allow the participant to preferentially choose between first- and third-person visual imagery to enhance potential clinical applicability. The difference in the way the participants were instructed to visualize may have attributed to the lower visual correlation between the questionnaires. Furthermore, functional movements may not be as challenging or complex for able-bodied individuals, compared to stroke survivors. Stroke survivors may need to image many steps of the same movement, for example, the MIQ-RS requests participants to reach forward, grasp a glass and lift it slightly off the table, then to replace the glass and return their hand to their lap or image their movements in relation to other parts of their body. In contrast, an able-bodied individual would just image the simple movement of their hand. For a stroke survivor a third person perspective (i.e., as though watching oneself on video) may be more efficacious.

In support of this argument, Hardy and Callow [24] suggest that for complex, form-based movements, athletes prefer a third-person (external) imagery perspective. A third-person perspective provides information that the athletes would not gain from a first-person (internal) perspective. For example, a diver must be able to imagine where their arm is in relation to their head, whether their feet are plantar-flexed, and so forth; a first-person imagery perspective would not provide this information. These complex athletic movements may be of equitable complexity to the stroke survivor's daily functional movements. Imaging these movements with a first person perspective may be inappropriate as it may not provide the individual with the information they seek from their imagery. Future research should examine the relationship between preferred imagery perspective and imagery ability and effectiveness in stroke populations.

 The presence of two significant outliers (>1.5 times the interquartile range [16]) for the average visual score in the able-bodied control group may be considered a limitation to this study. These outliers may be explained by possible increased interest and motivation felt by some individuals in the stroke group. Anecdotal observations provided by the examiner suggests that the healthy individuals may have been less attentive than the stroke survivors who may be more engaged due to increased personal investment and understanding of the importance of their contributions to stroke rehabilitation research. Imagery time may indicate whether participants are performing the mental tasks to the best of their ability. In this context, Sirigu et al. [25] has demonstrated that the time required to imagine a movement is correlated with the time taken to physically perform the same movement. The combination of mental chronometry and KVIQ has been proposed previously and has been used in two recent clinical studies [26, 27]. Future studies should investigate a possible correlation between motivation and imagery time.

The current study does not address interevaluator agreement. We assessed the reliability of the MIQ-RS using a single evaluator, which may have resulted in low variability and high test-retest reliability. This raises the question that if imagery ability using the MIQ-RS is rated by another evaluator, differences in scores could be obtained that may influence statistical conclusions from trial to trial. The MIQ-RS was designed to be administered in such a way that the task to be imagined is read to the volunteer word-for-word from a script and the volunteer (not the evaluator) records their score on a visual analog scale. Therefore, one can reasonably assume that any bias by the evaluator would have little if any effect on the score. However, this point poses a valid concern and follow-up experiments are being designed to address this question.

Neither the MIQ-RS nor the KVIQ-10 deal with the problem of assessing motor imagery in patients with lesions that may disrupt the capacity to perform imagery. Therefore, the claim that these tools are “valid” in all patients with stroke requires further exploration.

 In conclusion, among patients who had a stroke with mild-to-severe functional impairments and met the study inclusion criteria, the MIQ-RS is a valid and reliable outcome measure for motor imagery ability. The functional tasks included on the MIQ-RS may be a more valid representation of an individual's daily activities. This study found that scores on the FMA and MMSE were not associated with performance on the MIQ-RS. For future study, assessing the reliability and validity of the MIQ-RS in other neurologic populations (e.g., Parkinson's disease) may increase the questionnaire's clinical relevance. Assessing imagery ability is important as individuals with low imagery ability may benefit from alternative forms of rehabilitation or may need additional practice to improve their imagery ability with a resulting improvement in task performance.

## Figures and Tables

**Table 1:** Participant demographics.

	Stroke (*N* = 23)	Control group (*N* = 23)	*P*-value
Age (years)			
Mean	59.22	51	
SD	10.21	9.66	.008
Range	42–79	40–77	
Gender			
Male	*N* = 16	*N* = 12	.24
Female	*N* = 7	*N* = 11	
Handedness			
Right	*N* = 22	*N* = 22	1.0
Left	*N* = 1	*N* = 1	
Time elapsed since stroke (months)			
Mean	63.8	N/A	
SD	38.8		
Range	14–160		
Side of Stroke lesion			
Right	*N* = 8	N/A	
Left	*N* = 13		
Mini Mental Status Examination scores (MMSE)			
Mean	28.65	N/A	
SD	1.46		
Range	25–30		
Fugl-Meyer Assessment (FMA) UE motor score			
Mean	32.7	N/A	
SD	23.03		
Range	4–66		
MIQ-RS			
Mean 1st administration	5.62	5.29	
SD 1st administration	1.18	1.69	
Mean 2nd administration	5.65	5.28	
SD 2nd administration	1.19	1.57	
KVIQ-10			
Mean 1st administration	3.97	3.63	
SD 1st administration	0.80	1.11	
Mean 2nd administration	4.02	3.38	
SD 2nd administration	0.79	1.17	

SD: standard deviation; N/A: not applicable.

**Table 2:** Test-retest reliability of the MIQ-RS in the able-bodied control group.

Item number	Mean difference (SD)	*P*-value	ICC	95% CI
K1	.22 (1.13)	.37	.80	.58–.91
K3	−.61 (1.03)	.01	.86	.69–.94
K6	.09 (1.65)	.80	.64	.329–.83
K7	−.13 (1.18)	.60	.80	.58–.91
K9	−.17 (.83)	.33	.91	.80–.96
K11	−.22 (.90)	.26	.89	.75–.95
K12	.22 (.74)	.17	.89	.75–.95

**Average K**	−**.087 (.62)**	**.51**	**.94**	**.86**–**.97**

V2	.35 (.78)	.043	.89	.75–.95
V4	.17 (1.19)	.49	.89	.75–.95
V5	.26 (.81)	.14	.91	.80–.96
V8	.13 (.63)	.33	.95	.88–.98
V10	−.26 (.81)	.14	.91	.81–.96
V13	.13 (1.01)	.54	.91	.81–.96
V14	−.043 (.93)	.82	.90	.78–.96

**Average V**	**.11 (.27)**	**.074**	**.99**	**.98**–**.99**

SD: standard deviation; ICC: intraclass correlation coefficient; CI: confidence interval; K: kinesthetic item; V: visual item. Numeral represents item of the questionnaire.

**Table 3:** Test-retest reliability of the MIQ-RS in the stroke group.

Item number	Mean difference (SD)	*P*-value	ICC	95% CI
K1	−.26 (1.18)	.30	.77	.52–.89
K3	.13 (.97)	.53	.85	.67–.93
K6	−.04 (1.11)	.85	.78	.55–.90
K7	−.26 (1.32)	.35	.73	.46–.87
K9	−.13 (.97)	.53	.85	.68–.93
K11	.13 (1.10)	.58	.80	.59–.91
K12	−.087 (.79)	.60	.89	.76–.95

**Average K**	**−.075 (.63)**	**.58**	**.92**	**.83**–**.97**

V2	.087 (.73)	.58	.80	.59–.91
V4	−.17 (.78)	.30	.77	.54–.90
V5	.13 (.69)	.38	.78	.55–.90
V8	−.13 (.92)	.50	.65	.33–.83
V10	.17 (.89)	.36	.72	.44–.87
V13	.17 (1.07)	.45	.54	.18–.78
V14	−.22 (1.04)	.33	.59	.25–.80

**Average V**	**.0062 (.58)**	**.96**	**.83**	**.64**–**.92**

SD: standard deviation; ICC: intraclass correlation coefficient; CI: confidence interval; K: kinesthetic item; V: visual item. Numeral represents item of the questionnaire.

**Table 4:** Mean and standard deviation of the difference between measurements 1 and 2 with coefficient of repeatability (CR) for each of the measurements for the KVIQ-10 and MIQ-RS.

KVIQ-10	*N*	Mean difference	Std deviation	Coefficient of repeatability (SD × 1.96)
Able-bodied: kinesthetic	23	−0.2087	.40217	0.788
Able-bodied: visual	23	.0000	.39543	0.775
Stroke: kinesthetic	23	−0.0609	.57662	1.13
Stroke: visual	23	−0.0348	.47731	0.936

MIQ-RS	*N*	Mean difference	Std deviation	Coefficient of repeatability (SD × 1.96)

Able-bodied: kinesthetic	23	−0.0807	.61632	1.21
Able-bodied: visual	23	0.1056	.26974	0.527
Stroke: kinesthetic	23	−0.0745	.63285	1.24
Stroke: visual	23	0.0062	.58344	1.144

**Table 5:** Cronbach's alpha coefficients for MIQ-RS.

	Control + stroke	Control	Stroke
1st administration			
Kinesthetic	.97	.97	.97
Visual	.98	.98	.95

2nd administration			
Kinesthetic	.98	.98	.98
Visual	.98	.98	.95

**Table 6:** Correlation between scores on the MIQ-RS and the FMA and MMSE in the Stroke Group.

	Spearman correlation coefficient	*P*-value
FMA		
Kinesthetic scores	.40	.86
Visual scores	.24	.28
Total scores	.11	.61

MMSE		
Kinesthetic scores	−.32	.14
Visual scores	−.037	.87
Total Scores	−.24	.26

FMA: Fugl-Meyer Assessment; MMSE: Mini-Mental Status Examination.
